# P-1460. Judicious Use of Benzathine Penicillin G in Response to a Medication Shortage Alert

**DOI:** 10.1093/ofid/ofae631.1632

**Published:** 2025-01-29

**Authors:** William Campillo Terrazas, Rachel M Kenney, Amy Argyris, Anita Shallal, Michael Veve

**Affiliations:** Henry Ford Hospital, Detroit, Michigan; Henry Ford Hospital, Detroit, Michigan; Henry Ford Hospital, Detroit, Michigan; Henry Ford Health, Detroit, Michigan; Henry Ford Health, Detroit, Michigan

## Abstract

**Background:**

The national shortage of benzathine penicillin G (BPG) poses challenges in the treatment of syphilis. In response to this critical BPG shortage, our health system implemented a medication shortage alert within the electronic health record (EHR). The alert provides recommendations to optimize BPG utilization. This study investigates the impact of the BPG drug shortage on clinical practice.

**Figure:**
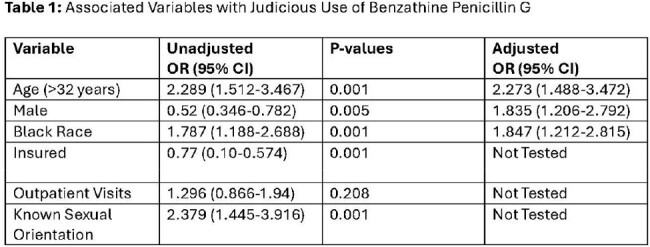

**Methods:**

This was an IRB-approved, retrospective cohort study focusing on patients >3 months who received BPG between 5/9/23–2/28/24. The study included inpatient and outpatient visits after implementing the medication shortage alert. Exclusions were applied for severe penicillin allergy, neurosyphilis, or congenital syphilis. Two cohorts were analyzed: the judicious BPG group (patients with primary, secondary, or latent syphilis receiving BPG), and the non-judicious group (patients receiving BPG for alternative diagnoses). The study assessed social determinants of health (SDOH) as primary outcomes and compared a separate cohort of syphilis patients receiving BPG or alternative therapy (e.g., doxycycline).

**Figure:**
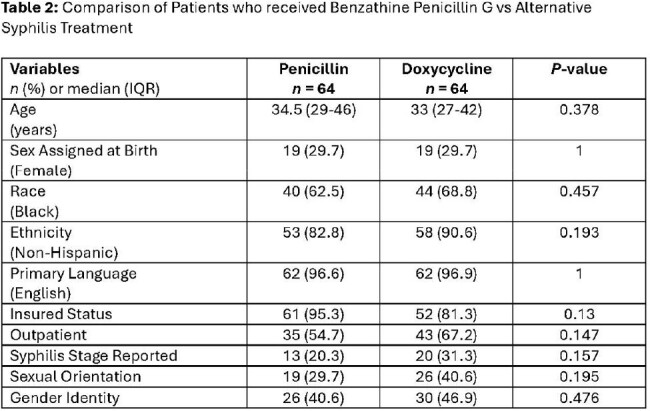

**Results:**

453 patients were included. Majority of patients were non-Hispanic Black (273, 60%) men (272, 60%), with a median age of 32 years (IQR: 22-44). Of these patients, 318 (70%) received judicious BPG, while 135 (30%) received non-judicious BPG. The most common non-judicious diagnosis was streptococcal pharyngitis (128, 95%). In multivariable logistic regression (Table 1), variables associated with judicious use included: age >32 years (adjOR: 2.273; 95% CI: 1.488-3.472), male sex at birth (adjOR: 1.835; 95% CI: 1.206-2.792), and black race (adjOR: 1.847; 95% CI: 1.212-2.815). Among a cohort of 128 syphilis patients who received either BPG (64, 50%) or doxycycline (64, 50%) treatment, those who received doxycycline were more likely to lack health insurance (35 [54.7%] vs. 43 [67.2%], p=0.15) and receive outpatient treatment (3 [4.7%] vs. 12 [18.7%], p=0.13) (Table 2). SDOH data were reported in < 50% of patient charts.

**Conclusion:**

Despite implementing an EHR drug shortage alert, 30% of BPG use was suboptimal and mostly for pharyngitis. Optimizing SDOH documentation represents an opportunity to assess health inequities and the impacts on patient outcomes for syphilis management.

**Disclosures:**

**Rachel M. Kenney, PharmD, BCIDP**, Medtronic Inc: Spouse is an employee, stockholder

